# Supplementary material for: Metabolic Singularities in Microsatellite-Stable Colorectal Cancer: Identifying Key Players in Immunosuppression to Improve the Immunotherapy Response
Source: Cancers (Basel). 2025 Feb 2;17(3):498. doi: 10.3390/cancers17030498 (PMC11815897; doi:10.3390/cancers17030498)
Supplement: Supplementary file 1 [file cancers-17-00498-s001.zip › Supplementary Table S1.pdf]

Supplementary Table S1. Prospective randomized clinical trials with PD1 inhibitors in esophageal, gastro-esophageal and gastric cancer.

Trial (Asian population, %)	N/ Location	Schedule	PFS HR (95% CI)	mPFS (E/C), months	2-y PFSR% (E/C)	OS HR (95% CI)	mOS (E/C), months	3-y OSR% (E/C)	ESMO-MCBS PFS/OS
CheckMate 649 (25%)	1581 (E,EG,G)	NIVO+CHT vs. CHT	0.79 (0.71–0.89)	7.7 (7–9.2) vs. 6.9 (5.6–6.9)	16 vs. 10	0.79 (0.71–0.88)	13.7 (6.7–19.1) vs. 11.6 (5.8–16.1)	17 vs. 10	1/2
ATTRACTION -04 (100%)	724 (EG/G)	NIVO+CHT vs. CHT	0.68 (0.51–0.90)	10.4 (8.4 14.7) vs. 8.3 (6.9–9.4)	30 vs. 20	0.90 (0.7–1.1)	17.4 (15.6–20.8) vs. 17.1 (15.1–19.6)	22 vs. 21	2/1
JAVELIN 100 (23%)	805 (EG/G)	AVE vs. CHT*	–	–	–	0.91 (0.74–1.11)	10.4 (9.1–12.0) vs. 10.9 (9.6–12.4)	22 vs. 15	NE/1
RATIONALE-305 (75%)	997 (EG/G)	TISLE+CHT vs. CHT	0.67 (0.55–0.83)	6.9 (5.7–7.2) vs. 6.2 (5.6–6.9)	17 vs. 9	0.80 (0.7–0.9)	15.0 (13.6–16.5) vs. 12.9 (12.1–14.1)	20 vs. 15	2/2
KEYNOTE-062 (25%)	507** (EG/G)	PEM+CHT vs. CHT	0.84 (0.70–1.02)	6.9 vs. 6.4	15 vs. 8	0.85 (0.70–1.03)	12.5 vs. 11.1	18 vs. 10	1/1
KEYNOTE-859 (34%)	1579 (EG/G)	PEM+CHT vs. CHT	0.76 (0.67–0.85)	6.9 (6.3–7.2) vs. 5.6 (5.5–5.7)	18 vs. 9	0.78 (0.70–0.87)	12.9 (11.9–14.0) vs. 11.5 (10.6–12.1)	21 vs. 12	1/2
KEYNOTE-590 (54%)	1020 (E)	PEM+CHT vs. CHT	0.65 (0.55–0.76)	6.3 vs. 5.8	10 vs. 0	0.73 (0.62–0.86)	12.4 vs. 9.8	25 vs. 18	2/3
ORIENT-16 (100%)	650 (EG/G)	SINTI+CHT vs. CHT	0.64 (0.52–0.77)	7.1 vs. 5.7	20 vs. 10	0.77 (0.63–0.94)	15.2 vs. 12.3	37 vs. 22*	2/2

mOS, median overall survival; mPFS, median progression-free survival; OSR, overall survival rate. * % were extrapolated from Kaplan-Meier curves.
